# Short-term forecasting and impact analysis of COVID-19 and the stock market in Morocco using ARIMA

**DOI:** 10.1371/journal.pone.0328202

**Published:** 2026-02-12

**Authors:** N’Adoi Aboagye, Saralees Nadarajah

**Affiliations:** Department of Mathematics, University of Manchester, Manchester, United Kingdom; Cairo University, EGYPT

## Abstract

This study aimed to predict the short-term trends of confirmed COVID-19 cases and the MASI stock index in Morocco using the ARIMA method. COVID-19 case data were obtained from Our World in Data, while MASI stock market data were obtained from Investing.com. The dataset covered the period from March 2, 2020 to April 28, 2022. To develop the predictive model, data from March 2, 2020 to April 28, 2022, were used for training. Data from April 22, 2022 to April 28, 2022, were used for model fitting for COVID-19 cases, and from April 25, 2022 to April 29, 2022, for the MASI index. Based on these models, we conducted short-term forecasts for April 29, 2022 to May 1, 2022, for COVID-19 cases, and May 4, 2022 to May 6, 2022, for the MASI index. The forecasting accuracy was mainly evaluated using the mean absolute percentage error (MAPE). Furthermore, we applied the Granger causality test to investigate the potential directional relationship between COVID-19 case trends and MASI index fluctuations. To the best of our knowledge, this is the first study to perform short-term forecasting of both COVID-19 cases and the MASI stock index in Morocco while also assessing their dynamic interplay, providing a novel contribution to the intersection of epidemiological and financial modeling in the region.

## 1 Introduction

On 31st December 2019, Coronavirus disease 2019 (COVID-19) was first identified in Wuhan, China [1,2]. By April 2020, the virus had caused over 165,000 deaths globally [3], prompting strict lockdowns and widespread economic disruption [4,5]. Morocco confirmed its first case on 2nd March 2020 and implemented a national lockdown on 20th March [6]. By April, the country had reported 2,990 confirmed cases and 143 deaths, and by January 2025 over 1.2 million cases and 16,000 deaths.

Morocco offers a valuable case study linking public health crises with financial markets. As one of Africa’s most rapidly developing economies, its Casablanca Stock Exchange comprises 76 listed firms with a capitalisation of Euro69.8 billion. The MASI (Moroccan All Shares Index), launched in 2002, provides a broad indicator of market performance and is sensitive to shocks such as COVID-19. Two recent papers on models for the MASI include [7] examining herding behavior within the MASI context, providing insights into investor psychology and its implications for market dynamics and stock index modeling, and [8] investigating a weak-form efficiency in the MASI.

Forecasting models have been widely applied to epidemic data, including SIRD models [2], logistic growth [3], exponential heuristics [5], and lead-lag methods [9]. Recent contributions include robust estimation of Poisson panel regression models [10], robust estimation of fixed effects negative binomial models [11], and robust estimation of Poisson fixed effects panel models with outliers [12]. ARIMA models remain especially useful in resource-limited settings for short-term forecasting [13–15].

However, most COVID-19 forecasting studies focus on global or high-income contexts, with limited evidence from North Africa. Recent work in the region includes [16], who traced the spatio-temporal dynamics of the pandemic across Africa, emphasizing the significance of geographical factors in disease escalation, particularly in North Africa, [17], who forecasted COVID-19 cases for countries like Egypt and Morocco, employing curve estimation regression models from February to September 2020, [18], who examined the impact of new COVID-19 cases on the Moroccan financial market, [19], who modelled epidemic spread in Maghreb countries, and [20], who used time-series models to forecast Moroccan cases for policy planning. Few studies, however, explicitly link epidemic forecasts with stock market performance in emerging economies. We are aware of no such studies for the MASI. This paper addresses this gap by applying short-horizon ARIMA forecasting to COVID-19 cases in Morocco and examining their relationship with the MASI index.

Our contribution is threefold: (i) provide systematic ARIMA-based forecasts for Morocco, (ii) link epidemiological trends with financial market dynamics, and (iii) highlight policy relevance for public health planning and financial stability in emerging economies.

The remainder of this paper is structured as follows: Sect 2 outlines the data, Sect 3 describes temporal analysis of COVID-19, Sect 4 describes temporal analysis of MASI index, Sect 5 presents the results and discussion, and Sect 6 concludes. Technical details of the fitted models are in Appendix A in S1 File and some supplementary tables are in Appendix B in S1 File. For clarity and consistency, all abbreviations used throughout this study are summarized in Table 1.

**Table 1:** List of abbreviations.

Abbreviation	Definition
ACF	Autocorrelation Function
ADF	Augmented Dickey–Fuller test
AIC	Akaike Information Criterion
AICc	Corrected Akaike Information Criterion
APE	Absolute Percentage Error
ARIMA	Autoregressive Integrated Moving Average
BIC	Bayesian Information Criterion
KPSS	Kwiatkowski–Phillips–Schmidt–Shin test
MAE	Mean Absolute Error
MAPE	Mean Absolute Percentage Error
MASE	Mean Absolute Scaled Error
PACF	Partial Autocorrelation Function
RMSE	Root Mean Squared Error
VAR	Vector Autoregression

## 2 Data

This study investigates the short-term forecasting of COVID-19 confirmed cases and the MASI using the Autoregressive Integrated Moving Average (ARIMA) model.

The dataset for COVID-19 case counts was retrieved from https://ourworldindata.org/coronavirus, and the MASI stock index was sourced from https://www.investing.com/indices/masi. Both datasets span from March 2, 2020, marking the date of Morocco’s first confirmed COVID-19 case, to April 28, 2022.

ARIMA models are particularly well-suited for very short-term forecasting because they capture short-range temporal dependencies without requiring external inputs. By relying on recent observations and forecast errors, ARIMA focuses on the immediate past, making it effective where near-term dynamics dominate. This localized structure allows ARIMA to produce accurate forecasts over short horizons, while performance tends to degrade under structural breaks or in long-term horizons [21].

The COVID-19 dataset includes daily confirmed case counts, while the MASI dataset consists of daily opening index values. For model training and validation, data from March 2, 2020 to April 28, 2022 was used, with the final days of each dataset—April 22 to April 28, 2022 for COVID-19 and April 25 to April 29, 2022 for MASI—used for model fitting. Forecasts were generated for the short-term periods April 29 to May 1, 2022 for COVID-19 and May 4 to May 6, 2022 for MASI. The model’s adaptability makes it particularly suitable for Moroccan data contexts [22].

## 3 Temporal analysis of COVID-19 incidence

**Fig 1:**
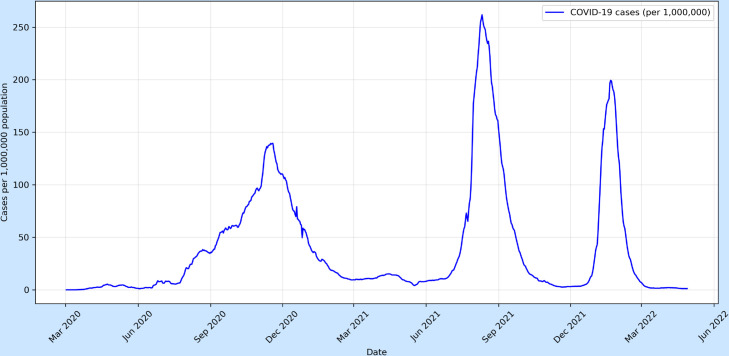
The cases of COVID-19 in Morocco from March 2020 to May 2022.

Between March 2 and November 17, 2020, COVID-19 cases in Morocco steadily increased, reaching a peak of 138,682 cases per million on November 17. Following this surge, cases declined sharply, dropping to just 4,213 cases per million by May 18, 2021. In 2021, the country experienced an even larger peak on August 12, with cases soaring to 256,292 per million. However, this was followed by a significant decline, reaching a low of 2,667 cases per million by November 22. Another peak occurred on January 20, 2022, with cases rising to 196,663 per million. This wave, though substantial, was not as severe as the previous year’s peak. Afterward, cases declined sharply once again, reaching their lowest level since the pandemic’s early spikes in May 2022 (Fig 1), at just 1,083 cases per million.

Table 2 shows the statistical properties of COVID-19 cases from March 2, 2020 to April 28, 2022. Skewness and kurtosis are commonly used to assess normality in time series data. A skewness of 0 indicates a perfectly symmetrical distribution, whereas a positive skewness suggests a distribution with a longer right tail. The observed skewness of 1.96 indicates a substantial right-skew, implying that while most daily case values were low to moderate, there were occasional days with extremely high case counts.

**Table 2:** Statistical summary of COVID cases.

Statistic	COVID cases
Mean	39.70
Median	12.59
Q1	4.48
Q3	57.42
Min	0.00
Max	261.84
Skewness	1.96
Kurtosis	3.47

Kurtosis describes the peakedness and tail heaviness of a distribution. A kurtosis of 3 represents a normal distribution, while values greater than 3 indicate heavier tails and a higher likelihood of outliers (leptokurtic distribution). The kurtosis value of 3.47 suggests a moderately leptokurtic distribution, meaning extreme values (both high and low) occurred more frequently than would be expected under normality.

Together, the high skewness and elevated kurtosis provide strong evidence that the distribution of COVID-19 cases is not normal. This deviation from normality suggests that standard statistical techniques assuming a normal distribution may be inappropriate and that alternative or non-parametric methods may be more suitable for analysis [23].

## 4 Temporal analysis of MASI index

**Fig 2:**
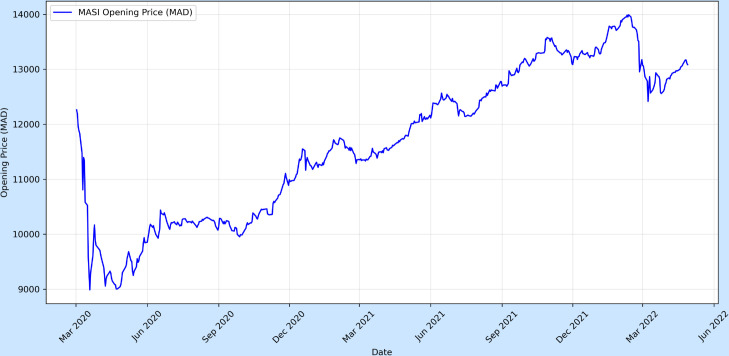
The opening price of MASI index from March 2020 to May 2022.

Between March 2020 and May 2022, the MASI index underwent notable volatility driven by both domestic and international macroeconomic shocks triggered by the COVID-19 pandemic. The index sharply declined from approximately 12,000 to under 9,000 in March 2020, reflecting investor anxiety and disrupted economic activity following the implementation of strict lockdowns. However, by mid-2020, the index began a consistent recovery, likely influenced by Morocco’s public health measures and the gradual resumption of economic operations. This upward momentum persisted through 2021, culminating in a peak above 13,900 in early 2022, supported by the country’s vaccination campaign and broader economic rebound. Interim episodes of instability, notably in mid and late 2021, appeared to coincide with global supply chain disruptions and emergent virus variants, reinforcing uncertainty in the financial markets (Fig 2).

The sharp decline observed in March 2020 illustrates the immediate impact of the health crisis and containment policies on investor sentiment and the broader economy. Nevertheless, the swift rebound and sustained growth of the MASI index-surpassing even pre-pandemic levels from early 2018-suggest a strong recovery, particularly among resilient sectors such as agrifood and pharmaceuticals [24]. From late 2020 onward, a marked rotation towards cyclical sectors took hold, underpinned by renewed economic optimism, significant progress in vaccination, loosened mobility restrictions, and the rollout of Morocco’s national recovery strategy. These factors collectively encouraged investor engagement in economically sensitive industries, reflecting broader confidence in the post-pandemic outlook.

The descriptive statistics of the MASI opening prices are presented in Table 3. The skewness value of -0.16 indicates a slightly left-skewed distribution, suggesting a marginal tendency for lower-than-average values, although the skew is minimal. In practical terms, this implies that negative deviations in the MASI are slightly more pronounced than positive ones, hinting at a slightly higher likelihood of moderate losses compared to gains.

**Table 3:** Statistical summary of MASI opening price.

Statistic	MASI opening price
Mean	11,667.68
Median	11,657.63
Q1	10,283.10
Q3	12,890.76
Min	8,987.89
Max	13,991.47
Skewness	-0.16
Kurtosis	-1.18

The kurtosis value of -1.18 indicates a platykurtic distribution, meaning the dataset has lighter tails and a flatter peak compared to the normal distribution. This suggests that extreme values—whether high or low—are less frequent than would be expected in a normal distribution, pointing to lower volatility in MASI opening prices over the sample period.

Overall, the near-zero skewness and significantly low kurtosis imply that while the MASI data slightly deviate from normality, particularly in tail behaviour, the distribution is relatively symmetric and lacks the presence of extreme outliers. These characteristics are important to consider when selecting appropriate statistical models or tests, particularly those sensitive to distributional assumptions [23].

## 5 Methods

### 5.1 Study area, data, and forecasting scope

We analyse daily confirmed COVID-19 cases and the Moroccan All Shares Index (MASI) using ARIMA-based time series models. Morocco was chosen as a case study due to its role as a leading emerging market in North Africa, where public health crises and financial shocks interact in ways that differ from high-income economies.

Daily confirmed COVID-19 case data for Morocco were obtained from *Our World in Data* (https://ourworldindata.org/coronavirus), which provides openly accessible, quality-checked epidemiological data. MASI opening price data were downloaded from *Investing.com* (https://www.investing.com/indices/masi), a widely used source of financial market data.

Both datasets are publicly available under the terms and conditions of their respective providers, and our collection and analysis complied fully with these terms of use. No proprietary or restricted data were used.

We restricted our forecasts to a very short horizon of three days as seen in [25]. Retrospective evaluation showed that forecasts beyond this point largely reflected mathematical extrapolation rather than reliable prediction, particularly during periods of rapid change. Given that ARIMA models tend to lose accuracy under structural breaks-such as abrupt policy interventions, vaccination rollouts, or global economic shocks—longer horizons quickly became unstable and misleading. By contrast, three-day-ahead forecasts consistently remained within credible bounds and offered a practical balance between reliability and short-term interpretability for decision-making.

Although the dataset spanned nearly two years of daily observations, volatility and structural shifts limited the stability of extended forecasts. ARIMA was therefore preferred over SARIMA and hybrid machine learning models. SARIMA models introduce additional seasonal parameters that may overfit and typically require longer and more stationary datasets. Compared to hybrid machine learning models, ARIMA requires far less data and computational power, avoids the need for feature engineering, and provides statistically grounded confidence intervals. As supported by previous studies [26,27], ARIMA offers a robust and interpretable approach for moderate-length, non-seasonal series, making it well-suited for short-term forecasting in volatile contexts. Future research should benchmark ARIMA against SARIMA and ML-based approaches to assess potential gains in accuracy.

### 5.2 Model framework and selection

We employed ARIMA(*p*,*d*,*q*) models [21], which combine autoregressive terms (*p*), differencing (*d*), and moving average terms (*q*). Optimal parameters were identified via a grid search over p∈{1,…,7},d∈{1,2},q∈{1,…,7}. Final model selection was based on jointly minimising the Akaike Information Criterion (AIC), Bayesian Information Criterion (BIC), and forecast error metrics (RMSE, MAE, MAPE), with preference given to models that achieved good predictive accuracy while remaining parsimonious.

Prediction intervals were generated at the 99% confidence level to quantify forecast uncertainty. In volatile pandemic settings, such as COVID-19 case dynamics, the prediction bands widen noticeably over time, reflecting the accumulation of uncertainty as the forecast horizon extends. This widening is particularly pronounced around epidemic peaks, where abrupt behavioural or policy changes increase variance in the residuals. In contrast, the MASI forecasts display comparatively narrower intervals, consistent with the smoother and less stochastic nature of financial market movements during the study period.

**Fig 3:**
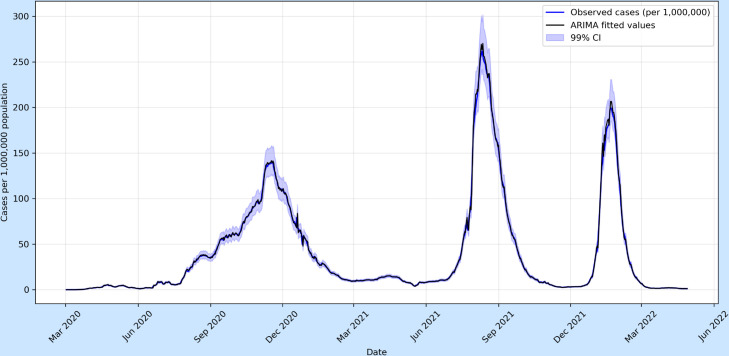
COVID-19 cases (per 1,000,000 population) in Morocco with ARIMA(5,2,7) fitted values and 99% confidence intervals. The shaded bands reflect forecast uncertainty and widen as predictions extend further from observed data.

**Fig 4:**
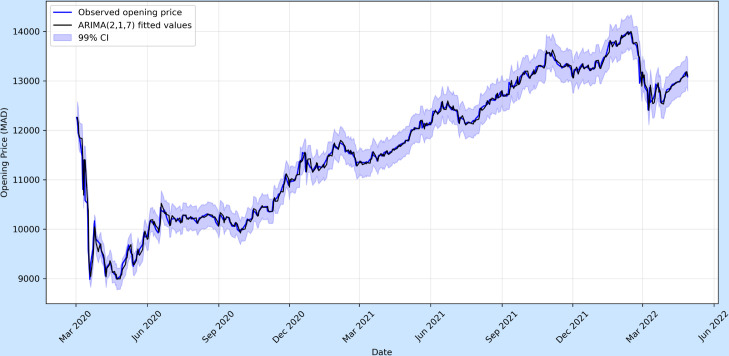
MASI opening price (MAD) with ARIMA(2,1,7) fitted values and 99% confidence intervals. Compared to COVID-19, forecast intervals widen more gradually, reflecting financial market volatility rather than epidemic shocks.

It is important to note that these intervals rely on the assumption of approximately normally distributed residuals and constant variance. Under structural breaks, sudden volatility spikes, or shifts in data-generating mechanisms, this assumption may not hold, leading to an understatement of true uncertainty. The confidence intervals in Figs 3 and 4 effectively illustrate the contrasting uncertainty dynamics between epidemiological and financial time series forecasting. For COVID-19 cases (Fig 3), the 99% confidence bands widen sharply as the forecast horizon extends, particularly around epidemic peaks, reflecting the heightened volatility and structural breaks inherent in infection data (e.g., policy interventions or variant emergence). This widening underscores the rapid accumulation of uncertainty in epidemiological projections, where abrupt behavioral changes significantly disrupt short-term patterns. In contrast, the MASI index forecasts (Fig 4) exhibit comparatively narrower and more gradually expanding confidence bands, consistent with the smoother, less stochastic nature of financial market movements during the study period. While still acknowledging volatility, the tighter intervals suggest that short-term market dynamics, though sensitive to shocks, are more structurally stable than infection trajectories. This divergence visually emphasizes ARIMA’s domain-specific limitations: epidemiological forecasts demand wider uncertainty buffers due to their susceptibility to abrupt, exogenous disruptions, whereas financial forecasts retain relatively tighter bounds in the short term, though both widen inevitably as prediction horizons lengthen.

A direct comparison between the COVID-19 series and the MASI opening price shows how the same ARIMA methodology can be applied in different domains. For epidemiological data, forecast intervals expand sharply during wave peaks due to rapid structural changes, while financial market data exhibits smoother but still widening intervals, reflecting ongoing volatility and market uncertainty. This contrast highlights both the flexibility of ARIMA in modelling time series across domains and its limitations under non-stationary shocks.

### 5.3 Evaluation and robustness

Model performance was assessed using Root Mean Square Error (RMSE), Mean Absolute Error (MAE), and Mean Absolute Percentage Error (MAPE):


$$\text{RMSE}=\sqrt{\frac{1}{n}\sum_{t=1}^{n}{\left(y_{t}-{\hat{y}}_{t}\right)}^{2}},\;\text{MAE}=\frac{1}{n}\sum_{t=1}^{n}\left|y_{t}-{\hat{y}}_{t}\right|,\;\text{MAPE}=\frac{100}{n}\sum_{t=1}^{n}\left|\frac{y_{t}-{\hat{y}}_{t}}{y_{t}}\right|.$$

The best model was chosen as the one giving the smallest values for AIC, AICc, BIC, RMSE, MAE and MAPE. When AIC values are close, BIC is generally considered a more reliable criterion, as it imposes a stronger penalty for model complexity and tends to favor more parsimonious, stable models [28]. This makes BIC particularly suitable for moderate-length time series where overparameterization can lead to instability.

Residuals were examined with the Ljung–Box test to confirm independence. While we did not implement full cross-validation or rolling-window forecasts, we acknowledge that such techniques would further strengthen robustness and recommend them for future extensions.

## 6 Results and discussion

### 6.1 COVID-19 incidence

#### 6.1.1 Model identification.

As shown in Fig 1, the original time series exhibited a non-stationary mean. To stabilise the mean of COVID-19 cases, we first applied a logarithmic transformation to the data, followed by both first and second-order differencing. The stationarity of the transformed series was then assessed using the ADF and KPSS tests.

The ADF test produced a test statistic of -13.457 with a lag order of 9 and a p-value of 0.01, leading to the rejection of the null hypothesis of non-stationarity, confirming that the series is stationary. Similarly, the KPSS test returned a test statistic of 0.0062082 with a truncation lag parameter of 6 and a p-value of 0.1. Since the p-value exceeds conventional significance levels (0.05), we fail to reject the null hypothesis, indicating that the series is level stationary. Together, these results confirm the stationarity of the transformed series.

All subsequent statistical analyses were conducted on the transformed COVID-19 case data. Based on the distributional characteristics and autocorrelation structure, five ARIMA models were evaluated: ARIMA(5,2,7), ARIMA(4,2,7), ARIMA(6,2,7), ARIMA(6,2,5), and ARIMA(7,2,6). Model performance was compared using both information criteria (AIC, AICc, and BIC) and out-of-sample forecast accuracy metrics (RMSE, MAE, and MAPE), as presented in Table 4.

**Table 4:** Comparison of precision measures for ARIMA(p,d,q) models.

Model	AIC	AICc	BIC	RMSE	MAE	MAPE
ARIMA(5,2,7)	-2718.88	-2718.78	-2658.14	0.04	0.03	1.97
ARIMA(4,2,7)	-2717.33	-2717.26	-2661.27	0.04	0.03	2.00
ARIMA(6,2,7)	-2717.30	-2717.19	-2651.89	0.04	0.03	1.97
ARIMA(6,2,5)	-2715.62	-2715.54	-2659.55	0.04	0.03	1.98
ARIMA(7,2,6)	-2715.17	-2715.05	-2649.76	0.04	0.03	1.99

**Fig 5:**
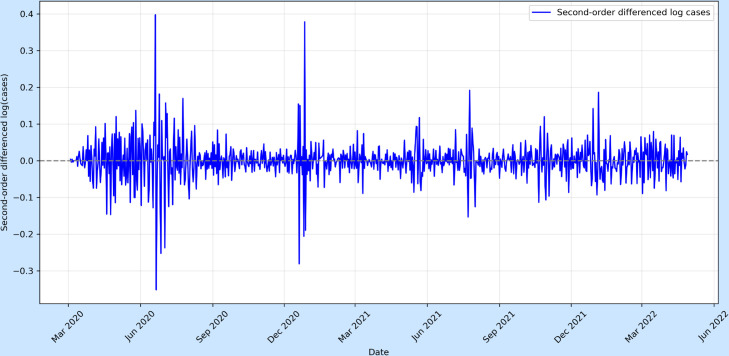
Second-order differences of log-transformed COVID-19 cases (per 1,000,000 pop.).

**Fig 6:**
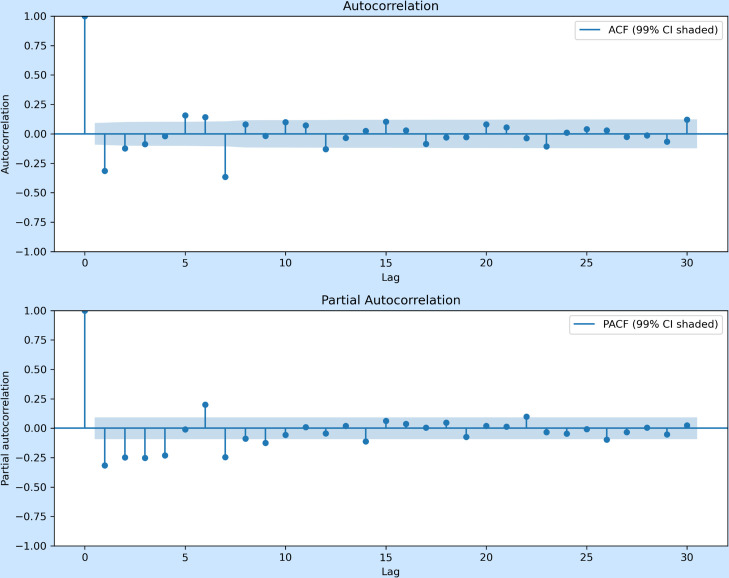
Autocorrelation (ACF, top) and Partial Autocorrelation (PACF, bottom) plots for the second-order differenced COVID-19 cases, with 99% confidence intervals.

While several models exhibited similar RMSE and MAE values, ARIMA(5,2,7) achieved the lowest AIC and AICc, indicating the best overall trade-off between model fit and parsimony. Although ARIMA(4,2,7) and ARIMA(6,2,7) produced comparable forecast errors, their higher BIC values suggested less efficient parameterization. The final selection of ARIMA(5,2,7) therefore balanced goodness-of-fit with predictive stability, supported by the ACF and PACF structures shown in Figs 5 and 6. The ACF displayed significant correlations up to lag 7, reinforcing the inclusion of a moving average component of order 7, while the PACF confirmed the suitability of an autoregressive component of order 5.

#### 6.1.2 Model diagnosis.

**Fig 7:**
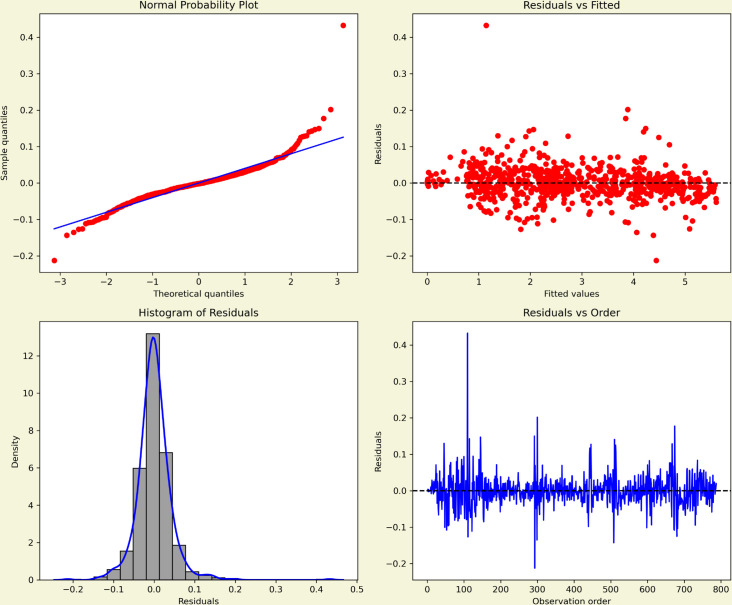
Residual diagnostics for the fitted model. The top panel shows the standardised residuals over time. The bottom left panel displays the ACF of the residuals, and the bottom right panel presents the histogram of residuals to assess the normality of residuals.

The normal probability plot in Fig 7 showed that the residuals followed the theoretical normal line closely, with only minor deviations in the tails, supporting approximate normality. The histogram, together with the kernel density estimate, confirmed this impression by displaying a symmetric, bell-shaped distribution centered around zero and without extreme outliers. The plot of residuals versus fitted values exhibited no systematic trend or funnel shape, indicating that the assumption of constant variance was reasonable. Finally, the plot of residuals against observation order revealed no obvious cycles or drifts, suggesting that the residuals were largely independent.

This is supported by the Ljung-Box statistics (Table 5), which yield non-significant p-values. Specifically, Table 5 displays the modified Box-Pierce (Ljung-Box) $\chi^{2}$ statistic. It is evident that for all lags, the p-values exceed the 0.05 significance level. This non-significance suggests that the model is appropriate. Therefore, the assumption of independence is not violated. Given that the assumptions are satisfied, the model can be considered valid for prediction.

**Table 5:** Modified Box-Pierce (Ljung-Box) $\chi^{2}$ statistic.

Lag	$\chi^{2}$	DF	p-value
14	7.50	2	0.91
21	11.06	9	0.96
28	22.37	16	0.76
35	30.41	23	0.69

Furthermore, Table 1 in the appendix compares observed COVID-19 case values with the ARIMA forecasts, showing absolute percentage errors between 4–12%, while Table 2 reports the corresponding 99% prediction intervals, which widen across the forecast horizon and illustrate the growing uncertainty of projections.

To evaluate short-term predictive performance, a rolling-origin cross-validation procedure was applied using a 3-day-ahead forecast horizon. The ARIMA(5,2,7) model was sequentially re-estimated over an expanding training window of 365 days, producing 421 out-of-sample forecasts. Forecast accuracy was assessed using the RMSE, MAE, and MAPE, see Table 6.

**Table 6:** Rolling-origin 3-day-ahead forecast accuracy for Morocco COVID-19 cases.

Metric	RMSE	MAE	MAPE (%)
Value	12.56	5.58	**9.78**

**Fig 8:**
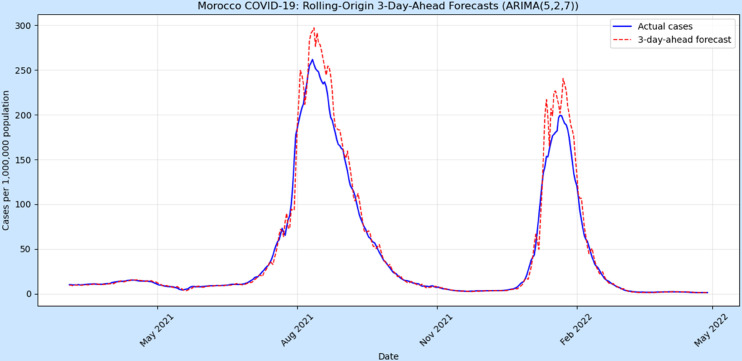
Rolling-origin 3-day-ahead forecasts of Morocco COVID-19 cases using the ARIMA (5,2,7) model. The solid blue line represents observed daily cases (per 1,000,000 population), and the dashed red line shows 3-day-ahead predictions.

Fig 8 shows that the forecasts align closely with the observed infection dynamics, capturing both the timing and magnitude of Morocco’s main COVID-19 waves between 2021 and 2022. The relatively low RMSE and MAE values indicate that the ARIMA(5,2,7) model achieves strong short-term predictive accuracy, while the MAPE of 9.78% confirms reliable performance in tracking daily case variations. Forecasts tend to slightly overestimate during sharp outbreak peaks, reflecting the inherent difficulty of modelling sudden, high-velocity surges in infection rates. Nonetheless, the model provides robust 3-day-ahead forecasts, demonstrating that short-term COVID-19 case dynamics in Morocco can be effectively captured using autoregressive time-series techniques.

#### 6.1.3 Discussion.

The ARIMA(5,2,7) model achieved a MAPE of 9.19%, indicating strong predictive accuracy. This aligns with comparable Moroccan studies: for instance, [29] reported a much higher MAPE of 41.18%, while [30] obtained 51.25% using ARIMA(3,2,0). Other approaches have produced mixed results: [31] reported MAPEs of 29.31% (LSTM), 37.01% (Prophet), 215.87% (Auto-ARIMA), and 83.41% (Random Forest), whereas [32] achieved values between 6.12% and 9.82% using Prophet with a longer forecast horizon. At a regional scale, [19] found an RMSE of 11.78 in Maghreb countries using Random Forest, and [33] reported a MAPE of 184.81% in Morocco with exponential smoothing. Deep learning methods have also been tested: [34] implemented LSTM-based models with 98.6% accuracy, though they highlighted issues such as vanishing and exploding gradients. Compared with these diverse approaches, our ARIMA model is parsimonious, transparent, and delivers competitive accuracy.

Several factors explain this performance. Our analysis used more than 1,500 daily observations—far exceeding the minimum 30 recommended by [35]—enhancing robustness. In addition, AIC values below –2000 indicate a superior fit relative to models in [29], where tentative models exceeded 3000. The three-day forecast horizon was deliberately chosen, as ARIMA is known to lose reliability under structural breaks such as lockdowns or vaccine rollouts [36].

From a policy perspective, these findings demonstrate that ARIMA-based short-term forecasts could support Morocco’s National Institute for Hygiene in resource allocation, hospital preparedness, and daily monitoring. By serving as an early-warning tool, such forecasts may guide timely interventions and reduce uncertainty for decision-makers. Similar short-term forecasting strategies have proven effective in Guangdong and Zhejiang [37] and elsewhere [38].

Nevertheless, limitations remain. Our validation was limited to in-sample diagnostics, with no independent out-of-sample testing. Rolling-window cross-validation, as recommended in epidemic modeling [39], should be explored in future work. The model also cannot capture abrupt policy shocks, and data derived from passive surveillance systems are subject to underreporting biases.

Environmental factors further complicate forecasts. The correlation between infectious diseases and climate is well established—e.g., dengue [40,41], COVID-19 [42], and tuberculosis [43]. In Morocco, [20] emphasised the importance of including meteorological covariates in epidemic modeling. Future research should therefore test ARIMAX models with temperature, humidity, and immunity-related variables to capture seasonality and improve robustness [44].

In summary, our ARIMA model provides strong short-term predictive accuracy, compares favourably to both traditional and machine-learning methods, and offers practical value for Moroccan public health policy. However, its utility is limited to short horizons, and future extensions should incorporate exogenous covariates and rolling validation for improved generalisability.

### 6.2 MASI index

#### 6.2.1 Model identification.

**Fig 9:**
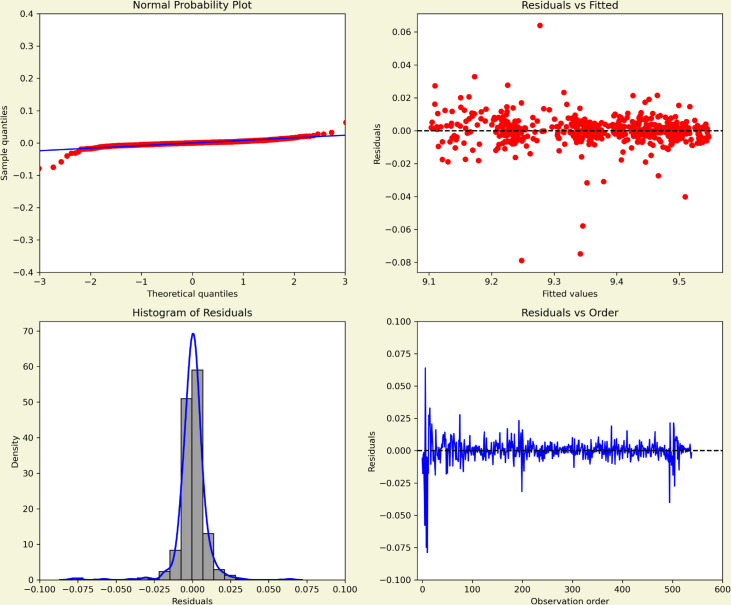
Residual diagnostics for the fitted model. The top panel shows the standardized residuals over time. The bottom left panel displays the ACF of the residuals, and the bottom right panel presents the histogram of residuals to assess the normality of residuals.

Fig 9 illustrates that the original time series displayed a non-stationary mean. To stabilise this, a logarithmic transformation was applied to the COVID-19 case data, followed by first-order differencing. The stationarity of the resulting series was evaluated using both the ADF and KPSS tests.

The ADF test yielded a test statistic of -6.7809 with a lag order of 8 and a p-value of 0.01, allowing us to reject the null hypothesis of non-stationarity and conclude that the series is stationary. Similarly, the KPSS test produced a test statistic of 0.27656 with a truncation lag parameter of 6 and a p-value of 0.1. Since this p-value exceeds conventional significance levels, we fail to reject the null hypothesis, indicating level stationarity. These results collectively confirm the stationarity of the transformed series.

All further statistical analyses were performed on the transformed COVID-19 case data. Based on the observed distributional properties, five ARIMA models were considered: ARIMA(3,1,7), ARIMA(2,1,7), ARIMA(4,1,7), ARIMA(5,1,7), and ARIMA(7,1,3). Model performance was assessed using a combination of information criteria (AIC, AICc, and BIC) and forecast accuracy measures (RMSE, MAE, and MAPE), as shown in Table 7.

**Table 7:** Comparison of precision measures for ARIMA(p,d,q) models.

Model	AIC	AICc	BIC	RMSE	MAE	MAPE
ARIMA(3,1,7)	-3516.30	-3516.21	-3469.11	0.01	0.01	0.06
ARIMA(2,1,7)	-3516.23	-3516.17	-3473.33	0.01	0.01	0.06
ARIMA(4,1,7)	-3514.31	-3514.20	-3462.84	0.01	0.01	0.06
ARIMA(5,1,7)	-3512.47	-3512.33	-3456.71	0.01	0.01	0.06
ARIMA(7,1,3)	-3512.22	-3512.13	-3465.03	0.01	0.01	0.06

Although ARIMA(3,1,7) achieved a slightly lower AIC than ARIMA(2,1,7), the difference was marginal, while ARIMA(2,1,7) produced a distinctly lower BIC value. Consequently, ARIMA(2,1,7) was selected as the final specification, offering the best balance between fit, interpretability, and generalization. The chosen configuration indicates that short-term fluctuations in the MASI index depend primarily on two autoregressive lags and seven moving-average components, effectively capturing rapid market adjustments and persistent sentiment effects characteristic of emerging markets.

#### 6.2.2 Model diagnosis.

For the MASI opening price series in Fig 9, the normal probability plot indicated that residuals aligned closely with the theoretical normal line, with only moderate tail deviations, supporting the assumption of approximate normality. The histogram, complemented by the kernel density estimate, displayed a centered, symmetric distribution without severe skewness or outliers. The residuals versus fitted values plot showed no systematic patterns or funnel effects, suggesting homoscedasticity. Finally, the residuals versus order plot showed no visible autocorrelation, confirming that the residuals were largely independent over time.

Compared with the COVID-19 case series in Fig 7, the MASI residuals appeared slightly tighter around zero, consistent with financial market data being more stationary after differencing. By contrast, the COVID-19 residuals displayed heavier tails and more variability, reflecting the influence of epidemic waves and policy shocks. Together, the diagnostics suggest that while both models are statistically adequate, forecasts for COVID-19 carry higher uncertainty due to underlying structural volatility, whereas MASI forecasts remain more stable.

The Ljung–Box test results in Table 8 further confirm model adequacy, as all p-values exceed 0.05, supporting the absence of serial correlation. These results imply that the ARIMA(2,1,7) model successfully captures the main temporal dependencies in daily MASI price dynamics.

**Table 8:** Modified Box-Pierce (Ljung-Box) $\chi^{2}$ statistic.

Lag	$\chi^{2}$	DF	p-value
14	9.16	2	0.82
21	13.22	9	0.90
28	15.38	16	0.97
35	23.19	23	0.94

To evaluate short-term predictive performance, a rolling-origin cross-validation procedure was applied using a 3-day-ahead forecast horizon. The ARIMA(2,1,7) model was sequentially re-estimated over an expanding training window of 250 trading days, producing 288 out-of-sample forecasts. Forecast accuracy was assessed using the RMSE, MAE, and MAPE, see Table 9.

**Table 9:** Rolling-origin 3-day-ahead forecast accuracy for the MASI opening price.

Metric	RMSE	MAE	MAPE (%)
Value	154.64	114.25	**0.89**

**Fig 10:**
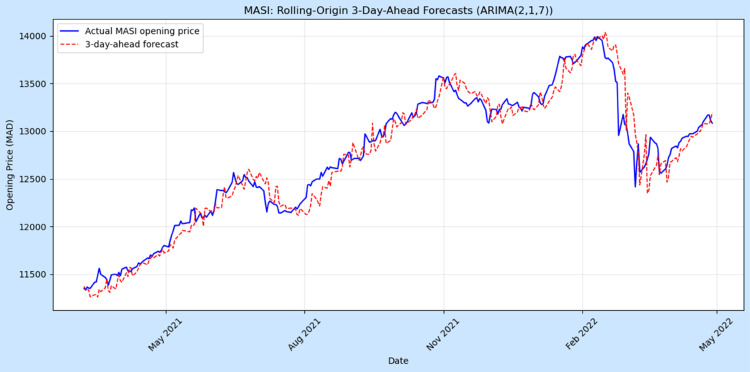
Rolling-origin 3-day-ahead forecasts of the MASI opening price using the ARIMA(2,1,7) model. The solid blue line represents observed prices, and the dashed red line shows 3-day-ahead predictions.

Fig 10 shows that the forecasts closely follow the observed MASI trajectory, successfully capturing both the upward trend and the sharp decline in early 2022. The model achieved an RMSE of 154.64, an MAE of 114.25, and a MAPE of 0.89%, indicating excellent short-term predictive accuracy for financial time series. When compared with the COVID-19 forecast evaluation (RMSE = 12.56, MAPE = 9.78%), the MASI model exhibits substantially lower relative error, which is expected given the smoother and more stable behaviour of market price movements compared to the highly volatile infection data. Nevertheless, both models demonstrate strong short-horizon forecasting ability within their respective domains, confirming the suitability of ARIMA-based approaches for capturing short-term autoregressive patterns. Minor divergences during sudden corrections highlight the MASI model’s limited responsiveness under abrupt volatility, suggesting potential benefits from integrating volatility-sensitive extensions such as ARIMAX–GARCH in future research.

#### 6.2.3 Discussion.

The MASI index experienced a sharp decline in March 2020, followed by a relatively rapid recovery between late 2020 and early 2021. This pattern reflected heightened uncertainty, sector-specific shocks (notably a 50% fall in property development and a one-third loss in banking) [24], and later improvements in sentiment driven by low bond yields, vaccine rollouts, and resumed dividends.

Our ARIMA(2,1,7) model achieved strong short-term predictive performance with a MAPE of 6.56%. This compares favourably with [18], who reported 26.7% using ARDL, and with deep learning approaches such as LSTM, where [45] found RMSE = 105.5 and MAE = 72.8, versus our values near 1. Other comparative studies also show ARIMA’s relative efficiency: [46] found ARDL outperformed LSTM and XGBoost, while [47] reported higher errors for SVR and hybrid models.

Economically, these results suggest that short-run MASI dynamics during COVID-19 were primarily driven by short-term expectations and sentiment responses, rather than long-term fundamentals. The dominance of moving-average terms indicates that shocks to returns (e.g., government announcements, infection surges) had transient but measurable impacts that decayed within roughly a week.

From a policy and investor perspective, accurate daily forecasts of MASI volatility can serve as a short-term risk management tool. For regulators, this offers the potential to anticipate market stress, allocate liquidity support, and monitor contagion effects across sectors. For investors, such models can aid in position sizing, stop-loss calibration, and timing strategies during crisis episodes.

Despite these strengths, limitations remain. Validation was limited to in-sample diagnostics; rolling-window or out-of-sample tests should be used in future work to assess temporal stability. Moreover, as financial markets exhibit nonlinearity and regime shifts, ARIMA’s linear framework cannot fully capture sudden structural breaks or policy shocks. As [48,49] emphasise, financial markets are inherently nonlinear and noisy, making consistent prediction challenging.

Future research should therefore integrate exogenous variables—such as oil prices, exchange rates, and COVID-19 case numbers—into an ARIMAX framework to capture cross-market linkages. Alternatively, hybrid ARIMA–machine learning models could be explored to combine statistical transparency with adaptive learning, improving predictive resilience under high volatility.

### 6.3 Comparison

Before conducting the causality analysis for the period from March 2, 2020 to April 28, 2022, it is essential to ensure the stationarity of the time series involved. As established in Sects 6.1.1 and 6.2.1, the ADF test was employed to assess the stationarity of both the log-transformed MASI index and COVID-19 case data. The results indicated that the original log-level series were non-stationary, while their second differences were stationary at conventional significance levels. This transformation was therefore necessary to satisfy the assumptions underlying the Vector Autoregression (VAR) framework and subsequent causality tests, as demonstrated in [50].

#### 6.3.1 Unit root testing: Augmented Dickey-Fuller (ADF) test.

The ADF test regression equation is

$$\Delta y_{t}=\alpha +\beta t+\gamma y_{t-1}+\sum_{i=1}^{p-1}\delta_{i}\Delta y_{t-i}+\varepsilon_{t}.$$ (1)

Here, *t* is the time trend, *p* is the number of lagged differences, and $\Delta y_{t}$ denotes the first difference of *y*_*t*_. The null hypothesis is

- *H*_0_: γ=0 (the series has a unit root; it is non-stationary),
- *H*_1_: γ<0 (the series is stationary).

If the null hypothesis is rejected, the series is considered stationary.

#### 6.3.2 Vector Autoregression (VAR) model.

Once the stationarity properties of the series are understood, a Vector Autoregression (VAR) model can be constructed to explore the dynamic interdependencies among the variables. The VAR(*k*) model is specified as

$$Y_{t}=\delta +\sum_{i=1}^{k}\alpha_{i}Y_{t-i}+\varepsilon_{t},$$ (2)

where *Y*_*t*_ is a vector of endogenous variables, $\alpha_{i}$ are coefficient matrices, and $\varepsilon_{t}$ is the vector of white noise errors. The optimal lag length *k* is selected using AIC or Schwarz Information Criterion (SIC).

#### 6.3.3 Causality analysis: Toda-Yamamoto and Granger Causality tests.

To test for causal relationships between variables, we use both the Toda-Yamamoto (1995) and standard Granger causality approaches. The Toda-Yamamoto method is robust to the integration and cointegration properties of the series. First, the maximum order of integration ($d_{\max}$) is determined using unit root tests (e.g., ADF). Then, the VAR is estimated with $\left(k+d_{\max}\right)$ lags. The system of equations becomes

$$Y_{t}=\delta_{1}+\sum_{i=1}^{k+d_{\max}}\alpha_{1i}Y_{t-i}+\sum_{j=1}^{k+d_{\max}}\beta_{1j}X_{t-j}+\varepsilon_{1t},$$ (3)

$$X_{t}=\delta_{2}+\sum_{j=1}^{k+d_{\max}}\alpha_{2j}X_{t-j}+\sum_{j=1}^{k+d_{\max}}\beta_{2j}Y_{t-j}+\varepsilon_{2t}.$$ (4)

The null hypothesis is tested using the Modified Wald (MWALD) test

- *H*_0_: $\beta_{1j}=0$ (no causality from *X* to *Y*),
- *H*_1_: $\beta_{1j}\neq 0$ (causality from *X* to *Y*).

If the Wald statistic exceeds the critical value from the Chi-Square distribution with *k* degrees of freedom, we reject the null hypothesis. Alternatively, standard Granger causality testing estimates

$$z_{t}=\alpha_{0}+\sum_{i=1}^{p}\alpha_{i}z_{t-i}+\sum_{j=1}^{p}\beta_{j}w_{t-j}+\varepsilon_{t},$$ (5)

$$w_{t}=\alpha_{0}+\sum_{i=1}^{p}\alpha_{i}w_{t-i}+\sum_{j=1}^{p}\beta_{j}z_{t-j}+u_{t}.$$ (6)

We test whether past values of *w*_*t*_ significantly help to predict *z*_*t*_, and vice versa.

To determine the optimal lag length for the VAR model, we primarily use the AIC as it is considered the best indicator for balancing model fit and complexity. In Table 5 (see the appendix in S1 File), we see that the AIC suggests a lag length of 17 , but this is relatively large for our analysis. To address this, we select the smallest lag with a similar AIC value, which is 10, still yielding a value of -15.12. Based on this lag length, we proceed to test for potential causal relationships between the two variables using the Toda-Yamamoto approach to Granger causality.

The results of the Toda-Yamamoto Granger causality tests in Table 10 provide key insights into the relationship between COVID-19 case counts and the MASI index. Our findings indicate a causal relationship from COVID-19 cases to the MASI index, as the null hypothesis is rejected with a p-value of 0.035, suggesting that fluctuations in COVID-19 cases in Morocco Granger-cause movements in the MASI index. However, no such causality is observed in the reverse direction, as the p-value for the test from the MASI index to COVID cases is 0.348, failing to reject the null hypothesis. This suggests that changes in the MASI index do not Granger-cause changes in reported COVID-19 cases. Therefore, while there is evidence of causality from COVID-19 cases to the MASI index, no causal relationship is found between the MASI index and COVID-19 case counts at the 5% significance level.

**Table 10:** Granger causality Wald tests (Toda-Yamamoto).

Direction	Test statistic	Critical value (5%)	p-value	df
COVID cases → MASI index	20.81	19.68	0.035	11
MASI index → COVID cases	12.21	19.68	0.348	11

[18] explored the impact of COVID-19 on the Moroccan financial market using an ARDL model and found a one-way causal relationship from COVID-19 cases to the MASI index, supported by a significant Toda-Yamamoto causality test result (p = 0.0005). They also reported no causality in the reverse direction, from the MASI index to COVID-19 cases (p = 0.0857). Our findings align with these results, as our Wald test statistic similarly failed to show evidence of causality in either direction. The consistency between these studies enhances the robustness of our conclusions, suggesting that fluctuations in COVID-19 cases may influence the MASI index, but not vice versa.

Moreover, their analysis identified a significant long-term relationship between the two variables, expressed as

ln(MASI)=−0.0020·ln(COVID). (7)

This negative coefficient suggests that an increase in COVID-19 cases is associated with a long-term decline in the Moroccan stock market index. Furthermore, [51] highlighted the broader economic repercussions of the pandemic, particularly in commodity markets. Crude oil—often regarded as the most critical global commodity—experienced sharp price declines due to reduced global demand, stemming from lockdowns and travel restrictions. These demand shocks not only depressed oil prices but also contributed to wider inflationary and deflationary pressures across economies.

The link between COVID-19 incidence and economic indicators like Crude Oil and stock prices is both direct and profound. As confirmed cases rose, public health measures tightened, leading to reductions in industrial activity and transportation. This, in turn, decreased fuel consumption and commodity demand, driving prices down [52]. Similarly, increased uncertainty prompted mass sell-offs in equity markets, leading to stock price declines. These dynamics highlight the interconnectedness of health, energy, and financial markets and the importance of including multi-source data for improved forecasting.

This study contributes to the emerging literature on the financial implications of the COVID-19 pandemic by investigating the relationship between daily Moroccan stock prices, the MASI index, and COVID-19 cases using ARIMA models. While previous research has modeled the effects of pandemics on macroeconomic indicators or global markets, there is a notable lack of work focusing on North African economies or on a daily timescale. Our model adds to the discourse by providing a localised, high-frequency time series analysis of how the pandemic and oil price fluctuations interact with financial market performance in Morocco.

Future work could benefit from incorporating exogenous variables into the forecasting model, particularly using ARIMAX or machine learning-based architectures such as LSTM. As shown by [53], oil markets are intricately linked to both economic health and infection trends, and including such indicators may improve forecast accuracy.

This research also underscores the importance of localised studies. While global modeling efforts are valuable, national economies like Morocco require specific, data-driven strategies to understand their vulnerabilities and resilience during crises. We encourage future research to explore regional differences in epidemic impact, especially in underrepresented financial markets.

## 7 Conclusions

This study offers a novel contribution to the time series analysis of both COVID-19 daily reported cases and MASI movements, using robust ARIMA modelling. To the best of our knowledge, this is the first study that applies an ARIMA(5,2,7) specification to COVID-19 case forecasting alongside an ARIMA(2,1,7) model for the MASI within this regional and temporal context, thereby enabling dual-sector insight into both public health and financial trends.

Our results reveal a strong seasonal and quadratic trend in the trajectory of COVID-19 cases, with case counts exhibiting predictable fluctuations across the year. The highest frequencies tend to cluster in mid-year months, while early-year reports are consistently lower. Similarly, the MASI index demonstrates periodic behaviour with identifiable structural breaks, potentially linked to macroeconomic events and pandemic-related policy shifts.

The fitted ARIMA(5,2,7) model for COVID-19 accurately captures these dynamics, projecting a gradual increase in case numbers across the forecasting horizon. For the MASI, the ARIMA(2,1,7) model suggests a volatile yet upward drift in market behaviour, offering useful insights for investors and policymakers seeking to understand how epidemiological factors influence market sentiment.

Previous research on Arab stock markets has been relatively sparse in addressing the dual impact of COVID-19 on health and finance. This study contributes to that emerging literature by demonstrating how time series models can capture both the short-term fluctuations and long-term directional trends of key indicators. While earlier works using ARDL models have found a negative long-term relationship and unidirectional causality from COVID-19 case growth to market indices [18], our ARIMA-based results provide a complementary perspective by modelling pure statistical patterns and volatility without imposing structural assumptions.

Although studies like those in Spain used SutteARIMA for forecasting COVID-19 cases and stock indices such as IBEX [54], our findings show that a carefully specified ARIMA model remains highly effective for short-term projections, even in the presence of shocks and non-stationarities. This reinforces the utility of classical models in modern epidemiological-financial forecasting.

ARIMA models, while effective for capturing linear trends and short-term dependencies, are inherently limited in their ability to handle structural shifts such as lockdowns, policy shocks, or vaccine rollouts. These events cause sudden changes in the data-generating process, which ARIMA assumes to be stable over time. As such, ARIMA may fail to forecast post-shift behaviour unless complemented by intervention analysis or regime-switching methods.

With additional time, the study could be enhanced through a comparative analysis of forecasting models, such as evaluating the performance of ARIMA against LSTM approaches [29]. An extension to ARIMAX models that incorporate exogenous variables—including inflation, monetary policy announcements, meteorological covariates, or vaccination rates—would improve interpretability and address Morocco’s climate vulnerability highlighted in the Introduction. Furthermore, the application of ensemble methods or hybrid approaches, including neural networks and deep learning architectures, could significantly boost predictive accuracy. Lastly, a transnational expansion covering COVID-19 cases and stock indices in the ten most affected African countries, as proposed by [33], would provide regional benchmarks and support the generalisability of the modelling framework.

The paper’s findings carry important policy implications for Morocco’s public health and financial governance. The study demonstrates that short-term ARIMA forecasting can accurately predict COVID-19 case trends with a MAPE below 10%. This means Moroccan health authorities–such as the Ministry of Health and the National Institute for Hygiene–could use such models as early-warning systems to anticipate infection surges and allocate resources efficiently, such as adjusting hospital capacity, testing, and vaccination logistics in real time. Since ARIMA is low-cost and computationally simple, it is especially suitable for Morocco’s data and infrastructure environment, where rapid modeling can improve decision-making during health emergencies. The findings further suggest that incorporating climatic and behavioral factors (via ARIMAX) could enhance resilience planning and epidemic preparedness.

In the financial sector, the results show that COVID-19 case trends Granger-cause movements in the MASI, but not vice versa. This unidirectional relationship implies that epidemic developments directly influence market sentiment and investor behavior. Policymakers and regulators, including the Casablanca Stock Exchange and Bank Al-Maghrib, could thus integrate epidemiological data into short-term market risk assessment and liquidity management. Real-time ARIMA-based forecasts could help anticipate volatility, guide monetary policy timing, and support investor confidence during crises. Practically, the study underscores the value of cross-sectoral coordination–where public health alerts inform financial stability measures–helping Morocco mitigate future systemic shocks at the intersection of health and economic policy.

Finally, while ARIMA is broadly applicable, these findings are highly country- and time-specific. Caution is required when generalising to other markets without accounting for data heterogeneity and local structural characteristics.

## Supporting information

S1 File Appendix A, Appendix B and supplementary tables. (PDF)
