# Supplementary material for: Short-term forecasting and impact analysis of COVID-19 and the stock market in Morocco using ARIMA
Source: PLoS One. 2026 Feb 12;21(2):e0328202. doi: 10.1371/journal.pone.0328202 (PMC12900332; doi:10.1371/journal.pone.0328202)
Supplement: S1 File — (PDF) [file pone.0328202.s001.pdf]

Technical details of time series models

This appendix briefly summarises the time-series models used in the study and collects the key formulas in one place. The main text focuses on an intuitive description of ARIMA; readers who are primarily interested in the results may skip these technical details, while those wanting the underlying mathematics can refer to this section.

A.1 White noise and stationarity

A weakly stationary time series has constant mean, constant variance, and autocovariance that depends only on lag (1; 2). White noise is a sequence of i.i.d. random variables with mean zero and constant variance (3). Informally, stationarity means that the overall behaviour of the series does not change over time, and white noise represents purely random "shock" terms with no predictable structure.

A.2 Autoregressive (AR) model

In an AR(p) model, the value of the time series at time t, denoted y_t, depends on its own previous p values:

$$y_t = \beta_0 + \beta_1 y_{t-1} + \beta_2 y_{t-2} + \cdots + \beta_p y_{t-p} + \varepsilon_t, \tag{1}$$

where ε_t is white noise. In other words, today's value is modelled as a weighted combination of past values plus a random error term.

A.3 Moving Average (MA) model

An MA(q) model expresses the current observation as a linear combination of white noise errors:

$$y_t = \mu + \varepsilon_t + \theta_1 \varepsilon_{t-1} + \cdots + \theta_q \varepsilon_{t-q}. \tag{2}$$

A.4 Autoregressive Moving Average (ARMA) model

An ARMA(p, q) model combines the autoregressive and moving-average components, allowing the series to depend both on its own past values and on past shocks. A process y_t follows ARMA(p, q) if:

$$\phi_p(B) y_t = \theta_q(B) a_t, \quad a_t \sim WN\left(0, \sigma^2\right). \tag{3}$$

Expanded form:

$$y_t = \beta_0 + \beta_1 y_{t-1} + \cdots + \beta_p y_{t-p} + \phi_1 \varepsilon_{t-1} + \cdots + \phi_q \varepsilon_{t-q} + \varepsilon_t. \tag{4}$$

This representation shows explicitly how both past values and past errors enter the model.

A.5 Autoregressive Integrated Moving Average (ARIMA) model

The ARIMA(p, d, q) model applies differencing d times to induce stationarity:

$$\phi_p(B)(1 - B)^d Z_t = \theta_q(B) a_t. \tag{5}$$

First and second differencing are:

$$y'_t = y_t - y_{t-1}, \quad y''_t = y_t - 2y_{t-1} + y_{t-2}. \tag{6}$$

The expanded ARIMA form is:

$$y_t^{(d)} = \beta_0 + \beta_1 y_{t-1}^{(d)} + \cdots + \beta_p y_{t-p}^{(d)} + \phi_1 \varepsilon_{t-1} + \cdots + \phi_q \varepsilon_{t-q} + \varepsilon_t. \tag{7}$$

Here, p controls how many past values of the differenced series are used (autoregressive part), d is the number of differences applied to remove trends or non-stationarity, and q controls how many past shocks are included (moving-average part). In practice, ARIMA models provide a flexible way to capture persistence, trends, and short-term noise in a single framework.

A.6 ARIMA with Exogenous Variables (ARIMAX)

ARIMAX extends ARIMA by including exogenous predictors:

$$y'_t = \beta_0 + \sum_{i=1}^{p} \beta_i y'_{t-i} + \sum_{j=1}^{q} \phi_j \varepsilon_{t-j} + \sum_{k=1}^{m} \theta_k X_{k,t} + \varepsilon_t, \tag{8}$$

where $X_{k,t}$ are exogenous variables with coefficients θ_k. This formulation allows external factors (such as climate variables or financial indicators) to enter the model alongside the internal time-series dynamics, making it particularly useful when outside drivers of the series are of substantive interest.

Supplementary tables

To improve clarity and reduce redundancy in the main text, several detailed tables have been moved to this section. They provide full numerical results for the ARIMA forecasts discussed in the Results and Discussion section, without interrupting the main narrative.

Table 1: Comparison of actual vs. ARIMA forecast for cases with APE.

Date	Actual	ARIMA forecast	APE
2022-04-22	1.18	1.32	0.12
2022-04-23	1.22	1.08	0.11
2022-04-24	1.24	1.37	0.10
2022-04-25	1.22	1.14	0.07
2022-04-26	1.17	1.31	0.12
2022-04-27	1.17	1.28	0.09
2022-04-28	1.22	1.26	0.04

Table 2: ARIMA forecast for cases with 99% prediction intervals.

Date	ARIMA forecast	Lower 99%	Higher 99%
2022-04-29	1.11	0.97	1.25
2022-04-30	1.09	0.88	1.30
2022-05-01	1.06	0.78	1.34

Table 3: Comparison of actual vs. ARIMA forecast for MASI with APE.

Date	Actual	ARIMA forecast	APE
2022-04-25	13169.67	12125.26	0.08
2022-04-26	13167.39	12274.78	0.07
2022-04-27	13106.89	12358.55	0.06
2022-04-28	13083.67	12298.60	0.06
2022-04-29	13134.64	12297.51	0.06

Table 4: ARIMA forecast for MASI with 99% prediction intervals.

Date	ARIMA forecast	Lower 99%	Higher 99%
2022-04-30	13151.02	13092.01	13210.02
2022-05-01	13084.87	12985.09	13184.66
2022-05-02	13045.18	12916.95	13173.41
2022-05-03	13083.32	12925.43	13241.20
2022-05-04	13141.12	12943.19	13339.05
2022-05-05	13116.26	12859.49	13373.04
2022-05-06	13067.29	12775.03	13359.54

Table 5: VAR order selection (* highlights the minimums).

Lag	AIC	BIC	FPE	HQIC
0	-3.74	-3.72	0.02	-3.73
1	-14.71	-14.66	0.00	-14.69
2	-14.91	-14.83	0.00	-14.88
3	-14.98	-14.87	0.00	-14.93
4	-15.06	**-14.92**	0.00	**-15.01**
5	-15.06	-14.88	0.00	-14.99
6	-15.07	-14.86	0.00	-14.99
7	-15.07	-14.82	0.00	-14.97
8	-15.10	-14.82	0.00	-14.99
9	-15.11	-14.80	0.00	-14.99
10	-15.12	-14.77	0.00	-14.98
11	-15.12	-14.74	0.00	-14.97
12	-15.11	-14.70	0.00	-14.95
13	-15.11	-14.66	0.00	-14.93
14	-15.11	-14.64	0.00	-14.93
15	-15.12	-14.61	0.00	-14.92
16	-15.12	-14.58	0.00	-14.91
17	**-15.12**	-14.55	**0.00**	-14.90
18	-15.11	-14.51	0.00	-14.88
19	-15.12	-14.48	0.00	-14.87
20	-15.11	-14.44	0.00	-14.84

References

[1] Peter J. Brockwell and Richard A. Davis. *Introduction to Time Series and Forecasting*. Springer, 2016.

[2] Douglas C. Montgomery, Cheryl L. Jennings, and Murat Kulahci. *Introduction to Time Series Analysis and Forecasting*. John Wiley and Sons, 2015.

[3] William W. S. Wei. *Time Series Analysis: Univariate and Multivariate Methods*. Addison-Wesley, 1994.
